# Induction chemotherapy followed by concurrent chemoradiation and nimotuzumab for locoregionally advanced nasopharyngeal carcinoma: preliminary results from a phase II clinical trial

**DOI:** 10.18632/oncotarget.13899

**Published:** 2016-12-10

**Authors:** Jian-feng Huang, Fu-zheng Zhang, Qin-zhou Zou, Le-yuan Zhou, Bo Yang, Jian-jun Chu, Jia-hua Yu, Hao-wen Zhang, Xiao-peng Yuan, Guo-mei Tai, Fen-ju Liu, C-M Charlie Ma

**Affiliations:** ^1^ Department of Radiobiology, School of Radiation Medicine and Protection and Jiangsu Provincial Key Laboratory of Radiation Medicine and Protection, Medical College of Soochow University, Collaborative Innovation Center of Radiation Medicine of Jiangsu Higher Education Institutions and School for Radiological and Interdisciplinary Sciences (RAD-X), Soochow University, Suzhou, China; ^2^ Department of Radiation Oncology, Affiliated Hospital of Jiangnan University, Wuxi, China; ^3^ Department of Radiation Oncology, Nantong Tumor Hospital, Affiliated Tumor Hospital of Nantong University, Nantong, China; ^4^ Department of Radiation Oncology, Fox Chase Cancer Center, Philadelphia, PA, USA

**Keywords:** nasopharyngeal carcinoma, nimotuzumab, chemoradiotherapy, induction chemotherapy

## Abstract

Overexpression of epidermal growth factor receptor can be found in more than 80% of patients with locoregionally advanced nasopharyngeal carcinoma and is associated with shorter survival. In this work, we evaluated the feasibility of adding nimotuzumab to chemoradiation in locoregionally advanced nasopharyngeal carcinoma. Twenty-three patients with clinically staged T3-4 or any node-positive disease were enrolled. They were scheduled to receive one cycle of induction chemotherapy followed by intensity-modulated radiotherapy, weekly administration of nimotuzumab and concurrent chemotherapy. Results showed that all patients received a full course of radiotherapy, 19(82.6%)patients completed the scheduled neoadjuvant and concurrent chemotherapy, and 22(95.7%) patients received =6 weeks of nimotuzumab. During the period of concurrent chemoradiation and nimotuzumab, grade 3-4 toxicities occurred in 14(60.9%) patients: 8 (34.8%) had grade 3-4 oral mucositis, 6(26.1%) had grade 3 neutropenia, and 1(4.3%) had grade 3 dermatitis. No acne-like rash was observed. With a median follow-up of 24.1 months, the 2-year progression-free survival and overall survival were 83.5% and 95.0%, respectively. In conclusion, concurrent administration of chemoradiation and nimotuzumab was well-tolerated with good compliance. Preliminary clinical outcome data appear encouraging with favorable normal tissue toxicity results comparing with historical data of concurrent chemoradiation plus cetuximab.

## INTRODUCTION

Concurrent chemoradiation(CCRT) is the standard combinational treatment modality for locoregionally advanced nasopharyngeal carcinoma(LA NPC).With the addition of platinum-based concurrent chemotherapy, a significant survival benefit has been achieved compared with radiotherapy alone [1, 2].However, up to 30% of patients still die of distant metastasis, while 10%-20% of patients will develop local and regional recurrences [3–5].The optimum sequence and combination of chemoradiotherapy for the treatment of LA NPC remains controversial. Newer treatment modalities, including different sequences and combinations of chemoradiotherapy are explored.

On the other hand, overexpression of EGFR can be found in more than 80% of patients with LA NPC [6]. High EGFR expression was associated with radiotherapy and chemotherapy resistance, and increased risks of locoregional recurrence, distant metastasis, and poor prognosis [7, 8]. Cetuximab, the most commonly used anti-EGFR antibody, has received considerable attention and achieved encouraging progress for the treatment of head and neck squamous cell carcinoma(HNSCC) [9–11]. The schedule of CCRT with cetuximab in LA NPC [12–15] has also demonstrated promising preliminary results. However, the incidence of acne-like rash and radiotherapy-related acute skin and mucosal toxic effects was significantly increased, thus its clinical application was greatly limited.

**Figure 1:**
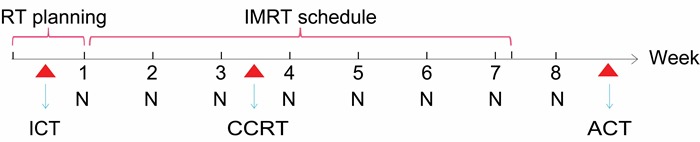
Multidisciplinary management of LA NPC in our study Abbreviations: LA NPC, Locoregionally advanced nasopharyngeal carcinoma; RT, Radiotherapy; IMRT, Intensity-modulated radiotherapy; ICT, Induction chemotherapy; CCRT, Concurrent chemotherapy; ACT, Adjuvant chemotherapy; N, Nimotuzumab

Distinct from cetuximab, nimotuzumab is a humanized EGFR monoclonal antibody with a unique safety profile [16]. However, there is little research focusing on the use of CCRT in combination with nimotuzumab in LA NPC. In the phase II single-arm trial presented here, we adopted a combinational treatment modality of induction chemotherapy, followed by concurrent chemoradiation and nimotuzumab for the treatment of LA NPC (Figure 1). The main purpose of the study is to evaluate the safety and treatment compliance of this treatment regimen. Here, we present the preliminary results of our study.

## RESULTS

### Patients and treatment compliance

From November 2011 to April 2016, 23 patients with a median age of 53 years (range, 23-73 years) were recruited into the study, baseline characteristics are listed in Table 1. All patients received one cycle of induction chemotherapy and a full course of intensity-modulated radiotherapy (IMRT) (Table 2). IMRT was interrupted in two patients who experienced severe side effects of grade 4 stomatitis (n = 1) and grade 3 dermatitis(n = 1). Nineteen(82.6%)patients completed the scheduled concurrent chemotherapy. In terms of compliance to nimotuzumab, 22 (95.7%) patients received ≥ 6 weeks of nimotuzumab, 19(82.6%)patients received 8 weeks of nimotuzumab. Nimotuzumab was discontinued in 4 patients due to refusal by patient (n = 3) and anaphylaxis (n = 1). Adjuvant chemotherapy was administered in 20(87%) patients: 18 received four cycles of adjuvant chemotherapy, 1 received three cycles, and another received two cycles.

**Table 1:** Baseline characteristics of patients

Characteristic	Number of patients
Total	23
Gender	
Male	18
Female	5
Age,years	
Median	53
Range	23-73
WHO histologic type	
II	18
III	5
Clinical T category	
T1	1
T2	7
T3	13
T4	2
Clinical N category	
N0	1
N1	5
N2	11
N3	6
UICC stage	
II	3
III	12
IVa	2
IVb	6

Abbreviations: WHO, World Health Organization; UICC, Union for International Cancer Control; T, tumor; N, lymph node

**Table 2:** Treatment compliance

Treatment compliance	Number of patients(%)
Induction chemotherapy	
received one cycle of induction chemotherapy	23(100)
Concurrent chemoradiation	
Radiotherapy	
received total dose of radiotherapy	23(100)
radiotherapy interruptions	2(8.7)
radiotherapy discontinuation	0(0)
Chemotherapy	
received one cycle of concurrent chemotherapy	19(82.6)
did not receive concurrent chemotherapy	4(17.4)
Nimotuzumab	
received eigtht weeks of nimotuzumab	19(82.6)
did not receive eigtht weeks of nimotuzumab	4(17.4)
received 7 weeks of nimotuzumab	1(4.3)
received 6 weeks of nimotuzumab	2(8.7)
received 1 weeks of nimotuzumab	1(4.3)
Adjuvant chemotherapy	
not scheduled to receive adjuvant chemotherapy	3(13.0)
scheduled to receive adjuvant chemotherapy	20(87.0)
received 4 cycles of adjuvant chemotherapy	18(78.4)
received 3 cycles of adjuvant chemotherapy	1(4.3)
received 2 cycles of adjuvant chemotherapy	1(4.3)

### Toxicity

No grade 3-4 toxicities occurred in the neoadjuvant setting. Adverse events from concurrent chemoradiation and nimotuzumab were listed in Table 3. The most common side effects were oral mucositis and neutropenia. Grade 3 or higher acute toxicities occurred in 14 (60.9%) of 23 patients: oral mucositis was recorded as grade 3 in 7 (30.5%) patients and grade 4 in 1 (4.3%) patient; 6(26.1%) patients suffered with grade 3 neutropenia, 3(13%) with grade 3 xerostomia, and 1 (4.3%) with grade 3 anemia. One case of grade 3 dermatitis was observed within the radiation field. Of the 19 patients who actually received concurrent chemotherapy, grade 3-4 oral mucositis, dermatitis and neutropenia occurred in 7(36.8%), 1(5.3%) and 5(26.3%) patients, respectively(Table 4). With respect to the toxicities of nimotuzumab, only 1 patient suffered anaphylaxis, no acne-like rash was found in any patients. In the adjuvant setting, the incidence rate of grade 3-4 acute toxicities in our cohort was 25% (5/20): 4(20%) patients with grade 4 neutropenia, and 1(5%) with grade 3 neutropenia.

**Table 3:** Adverse effects from concurrent chemoradiation and nimotuzumab

Adverse effects	Number of patients(%)		
	Grade 1–2	Grade 3	Grade 4
Neutropenia	11(47.8)	6(26.1)	0(0)
Thrombocytopenia	3(13.0)	0(0)	0(0)
Anemia	2(8.7)	1(4.3)	0(0)
Oral mucositis	15(65.2)	7(30.5)	1(4.3)
Xerostomia	20(87.0)	3(13.0)	0(0)
Dermatitis	19 (82.6)	1(4.3)	0(0)
Nimotuzumab-related rash	0(0)	0(0)	0(0)

**Table 4:** Grade 3-4 adverse effects in 19 patients who actually received concurrent chemoradiation and nimotuzumab

Grade 3-4 adverse effects	Number of patients(%)
Neutropenia	5(26.3)
Anemia	1(5.3)
Oral mucositis	7(36.8)
Xerostomia	2(10.5)
Dermatitis	1(5.3)

Xerostomia and hearing impairment were the most common late toxicities, and the degree decreased over time during follow-up. One patient experienced nasopharyngeal ulcer and bleeding, and recovered after symptomatic treatment. There were no cases of radiation-induced cranial nerve palsy, temporal lobe necrosis, osteonecrosis, myelopathy, or pituitary damage. There were no treatment-related deaths.

### Treatment response and survival

At the first 3-month follow-up after the completion of radiation, of the 23 patients, complete response (CR) and partial response (PR) of nasopharynx were achieved in 21(91.3%) and 2(8.7%) patients, respectively. Of the 22 patients with regional nodal involvement at diagnosis, 21 showed CR and 1 PR(Table 5). The residual tumor gradually disappeared or became less obvious at the following time.

**Table 5:** Response to treament (3months after radiotherapy)

Site	Number of patients(%)				
	CR	PR	SD	PD	CR+PR
Nasopharynx(n=23)	21(91.3%)	2(8.7%)	0(0)	0(0)	23(100%)
Regional lymph nodes(n=22)	21(95.5%)	1(4.5%)	0(0)	0(0)	22(100%)

Abbreviations: CR, Complete response; PR, Partial response; SD, Stable disease; PD, Progress disease

**Figure 2:**
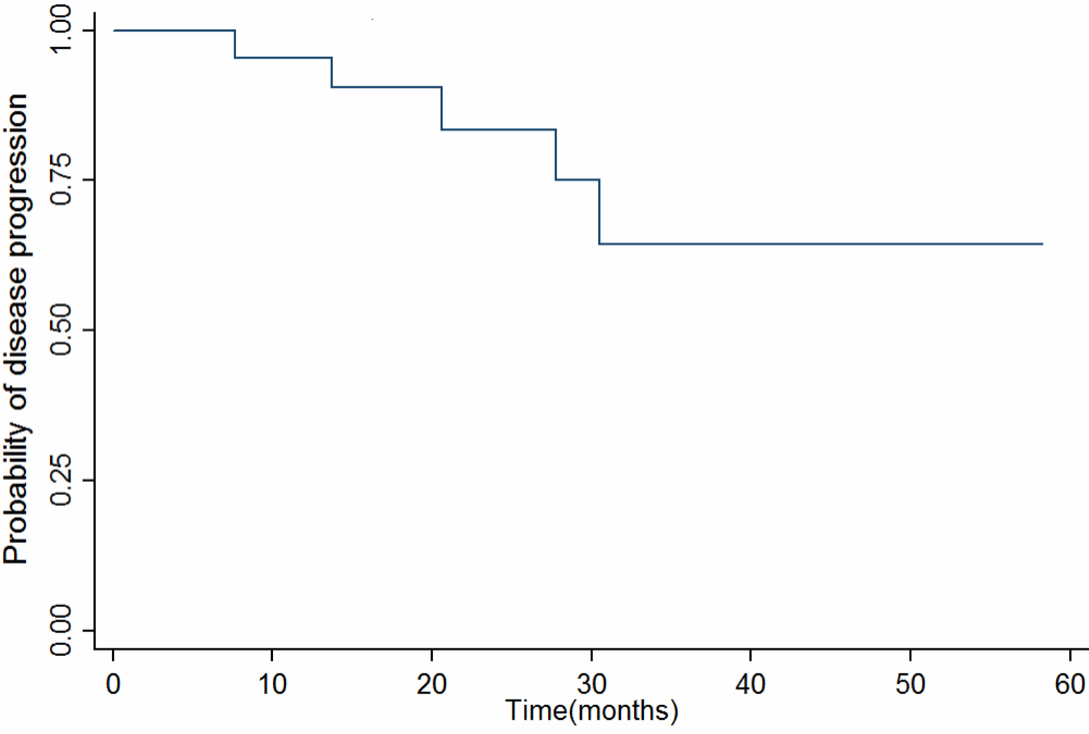
Kaplan–Meier survival estimate of PFS Abbreviations: PFS, Progression-free survival.

**Figure 3:**
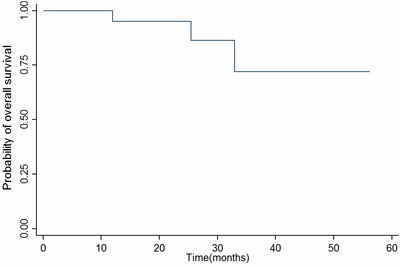
Kaplan–Meier survival estimate of OS Abbreviations: OS, Overall survival.

At a median follow-up of 24.1months [range, 5.0~58.2months], the estimated 2-year progression-free survival(PFS) was 83.5% (95% CI56.1% to 94.5%) (Figure 2), and the estimated 2-year overall survival(OS) was 95.0% (95% CI 69.5% to 99.3%)(Figure 3). At the last follow-up, a total of 5 patients failed with distant metastasis: 3 patients with bone metastases only, 1 with lung and bone metastases and 1 with liver and bone involvement, and three of them had died. Locoregional recurrence was not observed.

## DISCUSSION

The main objective of this phase II study is to determine the toxic effects and compliance of adding nimotuzumab to chemoradiation in LA NPC. Initial results show that this combinational treatment modality is feasible, with good compliance and acceptable toxicity. All patients received a full course of radiotherapy, 19(82.6%)patients completed the scheduled neoadjuvant and concurrent chemotherapy, and 22 (95.7%) patients received ≥ 6 weeks of nimotuzumab. No acne-like rash was observed. The incidence rate of grade 3-4 acute toxicities from concurrent chemoradiation and nimotuzumab in our cohort was 60.9 %.

As with other studies of CCRT, radiation-induced oral mucositis was the most common acute side-effect. In our study, Grade 3-4 oral mucositis occurred in 36.8% of patients who actually received CCRT+nimotuzumab. Compared with other studies of CCRT regimens, in which Grade 3-4 oral mucositis was reported between 29% and 52.4% [5, 17–19], the incorporation of nimotuzumab in our cohort was well-tolerated, without aggravation of oral mucositis. We also noted that 6(26.1%) patients suffered with grade 3 neutropenia, which was higher than that reported by a previous study [19]. Gastrointestinal side-effects, including anorexia, nausea and vomiting, were relatively mild in this study. We attribute this to the characteristic feature of nedaplatin, a derivative of cisplatin with less gastrointestinal toxicity but greater hematologic toxicity.

With the optimized local treatment by IMRT, locoregional recurrence rates have been markedly reduced [3–5]. In our study, no locoregional recurrence was observed. The main cause for failure in NPC is now distant metastases, especially in these with advanced disease at the primary site or regional lymph nodes. In the present study, 65.2 % and 73.9 % of patients developed T3-T4 and N2-N3 disease, respectively, and 5 patients developed one or more systemic diseases. However, our results should be regarded as preliminary because of the relatively small sample size and short follow-up.

At present, concurrent chemotherapy during the course of radiotherapy should be considered the standard of care for LA NPC. In addition to conventional 3-weekly (100mg/m2) or weekly (30-40mg/m2) schedule of cisplatin, other concurrent agents and regimes combined with radiotherapy have been explored. In a retrospective study by Xu and colleagues [20], the regime of paclitaxel plus nedaplatin was administered, comparable survival to those of the cisplatin-based regimens was found. Besides, considering that many patients with LA NPC demonstrate excessive expression of EGFR, and EGFR overexpression is associated with increased risks of distant metastasis and shorter survival, the strategy of EGFR inhibition seems attractive and has demonstrated encouraging clinical outcomes [21, 22]. However, evidence from published data is still insufficient to warrant changing the standard of care from chemoradiation to EGFR antagonists plus radiotherapy. In this preliminary study, we incorporated nimotuzumab into chemoradiation, the couplet regimen of docetaxel(75mg/m2) plus nedaplatin(80mg/m2) and weekly schedule of nimotuzumab were administered as concurrent agents. Concurrent chemotherapy was administered at intervals of 4 weeks after induction chemotherapy, which almost at the 4th week of radiotherapy. And then, during the next 3 weeks of radiotherapy, weekly nimotuzumab alone was administered.

As is well-known, cetuximab (a human-murine chimeric immunoglobulin G1 monoclonal antibody), is the first FDA approved agent in clinic against EGFR. The schedule of cetuximab in combination with CCRT in LA NPC has demonstrated promising preliminary results [12–15]. However, all of the studies reported an unexpectedly higher incidence of acneiform rash and grade 3/4 oral mucositis and dermatitis. Table 6 summarizes the observed adverse effects from these studies. Collectively, 85.8%-100% of patients experienced an acneiform rash, and grade 3/4 rash, mucositis and dermatitis occurred in 10%-39.4%, 66.7%-87% and 18.2%-25% of patients, respectively, which were much more common than those in our study. We ascribe these favorable normal tissue toxicity results to the feature of nimotuzumab. Compared with cetuximab, the affinity constant of nimotuzumab is lower. Nimotuzumab requires bivalent binding for stable attachment, which means that it selectively binds cells that express moderate to high EGFR levels, thus sparing normal tissues where EGFR expression is low [16]. Most recently, by a retrospective analysis [23], Liu and colleagues also concluded that nimotuzumab could be safely combined with concurrent chemoradiation in the treatment of LA NPC.

**Table 6:** Comparision of adverse effects with previously published studies treated with CCRT+cetuximab

	Study				
	Feng et al.	Ma et al.	Niu et al.	He et al.	Our series
Number of patients	28	30	33	21	23
Year	2014	2012	2013	2013	2016
All grades rash(%)	85.8	93	93.9	100	0
Grade 3/4 rash(%)	17.9	10	39.4	19.1	0
Grade 3/4 mucositis(%)	71.4	87	84.9	66.7	36.8
Grade 3/4 dermatitis(%)	25	20	18.2	23.8	5.3

Abbreviations: CCRT, Concurrent chemoradiation

The role of neoadjuvant or adjuvant chemotherapy remains ambiguous. The updated MAC-NPC meta-analysis confirmed that the addition of concomitant chemotherapy to radiotherapy significantly improves survival. However, this analysis does not completely answer the question whether there is a benefit of neoadjuvant or adjuvant chemotherapy in the concomitant setting [24]. There was no evidence of overall survival benefit observed with neoadjuvant or adjuvant chemotherapy. In addition, trends seen in specific trials showed that the additional treatment of neoadjuvant or adjuvant chemotherapy was poorly tolerated with increased toxic effects, and consequently, any possible benefit on survival may be offset [25, 26]. In our study, one cycle of induction chemotherapy was administered during the time period of IMRT planning within 1 week prior to radiotherapy, with an intention of reducing tumor volume and preventing early disease progression, subsequent delivery of one cycle of concurrent chemotherapy was administered at intervals of 4 weeks after induction chemotherapy. Adjuvant chemotherapy with the same regimen of neoadjuvant setting was not mandatory but was planned for selected patients (those with good tolerance to concurrent chemoradiation and good response to induction chemotherapy), with the intention of reducing treatment-related toxicities. He and colleagues reported a similar treatment modality of chemoradiation plus cetuximab, preliminary survival data are encouraging compared with historic data [14].

In summary, our study showed that concurrent administration of chemoradiation and nimotuzumab was well-tolerated and showed encouraging clinical activities. With a median follow-up of 24.1 months, the estimated 2-year PFS and OS were 83.5% and 95.0%, respectively, which appear promising in comparison with previous data [27, 28]. However, our results were only for a short follow-up and a small sample size. Further randomized, controlled, larger sample clinical trials are warranted.

## MATERIALS AND METHODS

### Patients

Patients were eligible for enrollment if they were aged 18 years or older, with Eastern Cooperative Oncology Group (ECOG) performance status 2 or lower, all of them had histopathologically confirmed World Health Organization (WHO) type II or III at the primary site. All patients were staged according to the Union for International Cancer Control(UICC) 2010 staging system. Patients were eligible for inclusion if their tumors showed evidence of T3 or T4 stage and any N stage, M0 or N1-3 stage and any T stage, M0, as assessed by clinical examination, nasopharyngoscopy, MRI scan of the nasopharynx and neck, chest computed tomography, abdominal sonography, and bone scan. MRI was recommended for local staging. Other inclusion criteria were no evidence of metastatic disease, adequate hematological, liver, and renal function. Conditions for exclusion included prior radiotherapy or chemotherapy, EGFR inhibitors, other cancers, pregnancy, lactation, or clinically significant cardiopulmonary diseases. This study was approved by the Institutional Ethics Committee of the affiliated hospital of Jiangnan university. All patients signed written informed consent before participating in the study.

### Treatment regimens

#### Radiotherapy

Intensity-modulated radiotherapy was delivered at 70Gy over 33 daily fractions to the planning target volume (PTV) of the primary tumor and metastatic cervical lymph nodes, 60Gy over 33 daily fractions to the subclinical disease around the primary tumor and upper neck, and 54Gy to the lower neck and supraclavicular region in the absence of nodal involvement. For patients with persistent lesions at the nasopharynx or regional lymph nodes at the end of IMRT, a radiation boost was given(4-6Gy in 2-3 fractions).All patients were treated once daily, five fractions weekly. In order to delineate the target accurately, computerized optimization was used with the help of fusion of MRI and CT images in the treatment planning system. Dose constraints to the critical organs at risk (OAR) were applied according to the Radiation Therapy Oncology Group(RTOG) 0225 protocol.

#### Chemotherapy and nimotuzumab therapy

A flow chart depicting the drug delivery of chemotherapy and nimotuzumab is shown in Figure 1. According to the protocol, all patients were scheduled to receive one cycle of induction chemotherapy during the time period of IMRT planning within 1 week prior to radiotherapy, subsequent delivery of concurrent chemotherapy with the same regimen of neoadjuvant setting was administered at intervals of 4 weeks after induction chemotherapy. Four cycles of adjuvant chemotherapy (with the same regimen of neoadjuvant setting) was not mandatory but was planned for selected patients (those with good tolerance to concurrent chemoradiation and good response to induction chemotherapy), starting at 4 weeks after radiotherapy. Detailed inclusion criteria for adjuvant chemotherapy were Eastern Cooperative Oncology Group (ECOG) performance status 2 or lower, nonexistence of grade 3-4 radiotherapy-related skin and mucosal toxic effects, and adequate haematological, liver, and renal function at 4 weeks after radiotherapy. Chemotherapy regimens: docetaxel 75mg/m2 on day 1, nedaplatin 80mg/m2 on day 2. Nimotuzumab was administered concomitantly with IMRT at a dose of 200mg weekly, commenced from the first day of radiotherapy. It was diluted in 250 mL saline and intravenously infused over 1 hour. Eight weeks of nimotuzumab was recommended but not mandatory.

#### Patient evaluation and follow-up

The assessment of tumor response used Response Evaluation Criteria for Solid Tumors(RECIST), which was performed 3months after the completion of radiotherapy with an MRI scan of the nasopharynx and neck. Evaluation of treatment-related toxicities began from the start of protocol therapy. Patients were monitored weekly during radiotherapy and before each chemotherapy cycle, with regard to hematologic and non-hematologic adverse effects. Systemic chemotherapy toxicities were assessed by the National Cancer Institute Common Toxicity Criteria (NCI CTCAE, version 3.0), whereas RT-related toxicities were graded according to the Acute and Late Radiation Morbidity Scoring Criteria of RTOG. Doses were modified in response to toxicities according to predefined guidelines.

Follow-up visits were at intervals of 3 months after radiotherapy for 2 years, 6 months for the next 3 years and annually thereafter. Assessments consisted of patient history, physical examination, MRI scan of the nasopharynx, chest computed tomography and abdominal sonography. Histological confirmation of locoregional recurrence and distant recurrence was encouraged.

#### Study end points and statistics

The main objective of the clinic trial was to evaluate the toxic effects and compliance of the combined modality treatment. Secondary endpoints included response rate to treatment, PFS and OS . OS was defined as the time from the date of enrollment to the date of death due to any cause or the date of lastfollow-up. PFS was the time between the date of enrollment and the first occurrence of locoregional or distant recurrence or the date of last follow-up. Locoregional recurrence was determined as the presence of recurrent disease at the nasopharynx and/or regional lymph nodes. Distant metastasis was defined as the presence of distant disease. Descriptive statistics [with 95% confi;dence intervals (CIs) where applicable] were used to report the study end points and survival curve was estimated with the Kaplan-Meier method.

## References

[1] Chan AT, Leung SF, Ngan RK, Teo PM, Lau WH, Kwan WH, Hui EP, Yiu HY, Yeo W, Cheung FY, Yu KH, Chiu KW, Chan DT (2005). Overall survival after concurrent cisplatin-radiotherapy compared with radiotherapy alone in locoregionally advanced nasopharyngeal carcinoma. J Natl Cancer Inst.

[2] Wee J, Tan EH, Tai BC, Wong HB, Leong SS, Tan T, Chua ET, Yang E, Lee KM, Fong KW, Tan HS, Lee KS, Loong S (2005). Randomized trial of radiotherapy versus concurrent chemoradiotherapy followed by adjuvant chemotherapy in patients with American Joint Committee on Cancer/International Union against cancer stage III and IV nasopharyngeal cancer of the endemic variety. J Clin Oncol.

[3] Lai SZ, Li WF, Chen L, Luo W, Chen YY, Liu LZ, Sun Y, Lin AH, Liu MZ, Ma J (2011). How does intensity-modulated radiotherapy versus conventional two-dimensional radiotherapy influence the treatment results in nasopharyngeal carcinoma patients?. Int J Radiat Oncol Biol Phys.

[4] Ng WT, Lee MC, Hung WM, Choi CW, Lee KC, Chan OS, Lee AW (2011). Clinical outcomes and patterns of failure after intensity-modulated radiotherapy for nasopharyngeal carcinoma. Int J Radiat Oncol Biol Phys.

[5] Sun X, Su S, Chen C, Han F, Zhao C, Xiao W, Deng X, Huang S, Lin C, Lu T (2014). Long-term outcomes of intensity-modulated radiotherapy for 868 patients with nasopharyngeal carcinoma: an analysis of survival and treatment toxicities. Radiother Oncol.

[6] Ma BB, Poon TC, To KF, Zee B, Mo FK, Chan CM, Ho S, Teo PM, Johnson PJ, Chan AT (2003). Prognostic significance of tumor angiogenesis, Ki 67, p53 oncoprotein, epidermal growth factor receptor and HER2 receptor protein expression in undifferentiated nasopharyngeal carcinoma--a prospective study. Head Neck.

[7] Sun W, Long G, Wang J, Mei Q, Liu D, Hu G (2014). Prognostic role of epidermal growth factor receptor in nasopharyngeal carcinoma: a meta-analysis. Head Neck.

[8] Zhang P, Wu SK, Wang Y, Fan ZX, Li CR, Feng M, Xu P, Wang WD, Lang JY (2015). p53, MDM2, eIF4E and EGFR expression in nasopharyngeal carcinoma and their correlation with clinicopathological characteristics and prognosis: A retrospective study. Oncol Lett.

[9] Bonner JA, Harari PM, Giralt J, Cohen RB, Jones CU, Sur RK, Raben D, Baselga J, Spencer SA, Zhu J, Youssoufian H, Rowinsky EK, Ang KK (2010). Radiotherapy plus cetuximab for locoregionally advanced head and neck cancer: 5-year survival data from a phase 3 randomised trial, and relation between cetuximab-induced rash and survival. Lancet Oncol.

[10] Koutcher L, Sherman E, Fury M, Wolden S, Zhang Z, Mo Q, Stewart L, Schupak K, Gelblum D, Wong R, Kraus D, Shah J, Zelefsky M (2011). Concurrent cisplatin and radiation versus cetuximab and radiation for locally advanced head-and-neck cancer. Int J Radiat Oncol Biol Phys.

[11] Rowan K (2010). Should cetuximab replace cisplatin in head and neck cancer?. J Natl Cancer Inst.

[12] Ma BB, Kam MK, Leung SF, Hui EP, King AD, Chan SL, Mo F, Loong H, Yu BK, Ahuja A, Chan AT (2012). A phase II study of concurrent cetuximab-cisplatin and intensity-modulated radiotherapy in locoregionally advanced nasopharyngeal carcinoma. Ann Oncol.

[13] Niu X, Hu C, Kong L (2013). Experience with combination of cetuximab plus intensity-modulated radiotherapy with or without chemotherapy for locoregionally advanced nasopharyngeal carcinoma. J Cancer Res Clin Oncol.

[14] He X, Xu J, Guo W, Jiang X, Wang X, Zong D (2013). Cetuximab in combination with chemoradiation after induction chemotherapy of locoregionally advanced nasopharyngeal carcinoma: preliminary results. Future Oncol.

[15] Feng HX, Guo SP, Li GR, Zhong WH, Chen L, Huang LR, Qin HY (2014). Toxicity of concurrent chemoradiotherapy with cetuximab for locoregionally advanced nasopharyngeal carcinoma. Med Oncol.

[16] Ramakrishnan MS, Eswaraiah A, Crombet T, Piedra P, Saurez G, Iyer H, Arvind AS (2009). Nimotuzumab, a promising therapeutic monoclonal for treatment of tumors of epithelial origin. MAbs.

[17] Wolden SL, Chen WC, Pfister DG, Kraus DH, Berry SL, Zelefsky MJ (2006). Intensity-modulated radiation therapy (IMRT) for nasopharynx cancer: update of the Memorial Sloan-Kettering experience. Int J Radiat Oncol Biol Phys.

[18] Kam MK, Teo PM, Chau RM, Cheung KY, Choi PH, Kwan WH, Leung SF, Zee B, Chan AT (2004). Treatment of nasopharyngeal carcinoma with intensity-modulated radiotherapy: the Hong Kong experience. Int J Radiat Oncol Biol Phys.

[19] Xu T, Zhu G, He X, Ying H, Hu C (2014). A phase III randomized study comparing neoadjuvant chemotherapy with concurrent chemotherapy combined with radiotherapy for locoregionally advanced nasopharyngeal carcinoma: updated long-term survival outcomes. Oral Oncol.

[20] Xu J, He X, Cheng K, Guo W, Bian X, Jiang X, Zhang L, Huang S (2014). Concurrent chemoradiotherapy with nedaplatin plus paclitaxel or fluorouracil for locoregionally advanced nasopharyngeal carcinoma: Survival and toxicity. Head & neck.

[21] Huang XD, Yi JL, Gao L, Xu GZ, Jin J, Yang WZ, Lu TX, Wu SX, Wu RR, Hu WH, Xie WC, Han F, Gao YH (2007). Multi-center phase II clinical trial of humanized anti-epidermal factor receptor monoclonal antibody h-R3 combined with radiotherapy for locoregionally advanced nasopharyngeal carcinoma. Zhonghua Zhong Liu Za Zhi.

[22] Zhai RP, Ying HM, Kong FF, Du CR, Huang S, Zhou JJ, Hu CS (2015). Experience with combination of nimotuzumab and intensity-modulated radiotherapy in patients with locoregionally advanced nasopharyngeal carcinoma. Onco Targets Ther.

[23] Liu ZG, Zhao Y, Tang J, Zhou YJ, Yang WJ, Qiu YF, Wang H (2016). Nimotuzumab combined with concurrent chemoradiotherapy in locally advanced nasopharyngeal carcinoma: a retrospective analysis. Oncotarget.

[24] Blanchard P, Lee A, Marguet S, Leclercq J, Ng WT, Ma J, Chan AT, Huang PY, Benhamou E, Zhu G, Chua DT, Chen Y, Mai HQ (2015). Chemotherapy and radiotherapy in nasopharyngeal carcinoma: an update of the MAC-NPC meta-analysis. The Lancet. Oncology.

[25] Tan T, Lim WT, Fong KW, Cheah SL, Soong YL, Ang MK, Ng QS, Tan D, Ong WS, Tan SH, Yip C, Quah D, Soo KC (2015). Concurrent chemo-radiation with or without induction gemcitabine, Carboplatin, and Paclitaxel: a randomized, phase 2/3 trial in locally advanced nasopharyngeal carcinoma. International journal of radiation oncology, biology, physics.

[26] Lee AW, Tung SY, Chua DT, Ngan RK, Chappell R, Tung R, Siu L, Ng WT, Sze WK, Au GK, Law SC, O’Sullivan B, Yau TK (2010). Randomized trial of radiotherapy plus concurrent-adjuvant chemotherapy vs radiotherapy alone for regionally advanced nasopharyngeal carcinoma. Journal of the National Cancer Institute.

[27] Chan AT, Teo PM, Ngan RK, Leung TW, Lau WH, Zee B, Leung SF, Cheung FY, Yeo W, Yiu HH, Yu KH, Chiu KW, Chan DT (2002). Concurrent chemotherapy-radiotherapy compared with radiotherapy alone in locoregionally advanced nasopharyngeal carcinoma: progression-free survival analysis of a phase III randomized trial. Journal of clinical oncology.

[28] Lee AW, Lau WH, Tung SY, Chua DT, Chappell R, Xu L, Siu L, Sze WM, Leung TW, Sham JS, Ngan RK, Law SC, Yau TK (2005). Preliminary results of a randomized study on therapeutic gain by concurrent chemotherapy for regionally-advanced nasopharyngeal carcinoma: NPC-9901 Trial by the Hong Kong Nasopharyngeal Cancer Study Group. J Clin Oncol.

